# Personalizing prognostic prediction in early-onset Colorectal Cancer

**DOI:** 10.7150/jca.46871

**Published:** 2020-09-25

**Authors:** Jian Liu, Zhengru Liu, Jiao Li, Shan Tian, Weiguo Dong

**Affiliations:** Department of Gastroenterology, Renmin Hospital of Wuhan University, Wuhan University, Wuhan, Hubei, 430060, China.

**Keywords:** Early-onset colorectal cancer, Nomogram, TNM stage, Prognosis, Overall survival

## Abstract

Accurately estimating prognosis based on clinicopathologic variables could improve risk stratification for patients with early-onset colorectal cancer (EOCRC). Our primary goal was to create and validate a survival nomogram with adequate performance for predicting overall survival (OS) in patients with EOCRC. Least absolute shrinkage and selection operator (LASSO) Cox regression analysis was applied to identify clinical features statistically related to OS. Then we established and internally validated a survival nomogram based on surveillance, epidemiology and end results (SEER) database (N=23813). A cohort of 77 patients with EOCRC from Renmin Hospital of Wuhan University (RHWU) was employed to detect the external validity of the survival nomogram. Moreover, we compared the predictive accuracy of survival nomogram with TNM stage, and also compared the OS between endoscopy and surgery groups before and after propensity score matching (PSM) among EOCRC patients with early stage (Tis-T1N0M0). We selected seven informative indexes (N stage, M stage, perineural invasion, chemotherapy, surgery primary site, summary stage and tumor grade) for the construction of the survival nomogram. Then the survival nomogram exhibited good discrimination with C-index of 0.829, 0.841 and 0.796 in the SEER training, SEER validation and RHWU validation sets, respectively. Calibration curves showed good concordance between the survival nomogram predictions and actual outcomes for 1-year, 3-year and 5-year OS. Furthermore, the survival nomogram was superior to risk stratification by TNM stage in predicting OS among patients with EOCRC. Early-stage patients treated with endoscopy showed similar survival to those with surgery before and after PSM. We proposed a survival nomogram based on the extensively used parameters to precisely predict OS in EOCRC patients. This survival nomogram will contribute to aid oncologists better risk stratification and prognostication for patients with EOCRC.

## Introduction

Colorectal cancer (CRC) has become the second most common cause of cancer-related death in the United States 1. About 147,950 individuals will be newly diagnosed with CRC and 53,200 patients will die from CRC in 2020 1. The overall morbidity and mortality trends of CRC have evolved dramatically in recent years 2,3. Due to the early detection of CRC with stool-based tests or colonoscopy, the incidence and mortality rates decreased among patients over the age of 50 4,5. However, recent studies have drawn attention to a slight increase both in the incidence and mortality rates of CRC in patients earlier than 50 years 6,7. The incidence of CRC in younger patients increased from 0.01% in 2004 to 0.013% in 2016, and its mortality rate rose from 2.53 per 100,000 in 2004 to 2.97 per 100,000 in 2016 6. Moreover, the incidence of CRC in young patients is predicted to increase by as much as 142% by 2030 8,9. Unfortunately, there is no simple explanation for such an increase both in the incidence and mortality rates among younger patients with CRC 10.

Early-onset colorectal cancer (EOCRC) is a special subtype of CRC and our knowledge related to the etiology and mechanism of this subtype is far from being comprehensive 10-14. EOCRC is defined as CRC diagnosed under the age of 50 with certain hereditary predisposition, which has distinct clinicopathological and molecular features compared with traditional CRC 15,16. Mounting evidence 9,11,15,16 has demonstrated that EOCRC presents at a later TNM stage and possess a more aggressive histopathology. Most studies focused on the rising incidence of EOCRC from the Surveillance Epidemiology and End Results (SEER) database, but no current study focused attention on the potential factors affecting survival of EOCRC. Accurate estimation of survival among patients with cancer is always difficult, even for some experienced physicians 17. A more precise estimation of survival tailored to individual patient with EOCRC is a potentially practical tool for clinicians. Nomogram is a kind of statistic tool that combines all significant prognostic indexes and represents with a simple graphical model 18,19. Hence, creating a survival nomogram based on the key clinical parameters with good discrimination and calibration abilities is critical to improve the prognosis of patients with EOCRC.

In this present study, we aimed to establish and verify a survival nomogram integrating the accessible clinical features to improve prognostication for patients with EOCRC in clinical practice. We initially used LASSO Cox regression to screen variables showing both statistical and clinical significance in the SEER training set. Subsequently, the informative parameters were further integrated into the establishment of survival nomogram, and concordance index (C-index) was employed to evaluate the predictability of this nomogram in the SEER training and validation sets. Then, the predictive value of the survival nomogram was also validated in Renmin Hospital of Wuhan University (RHWU) validation set. Finally, we compared the predictive performance of the prognostic nomogram with TNM staging system which is extensively applied for accurate prognostication in clinical practice.

## Materials and Methods

### Study population

**Figure 1:**
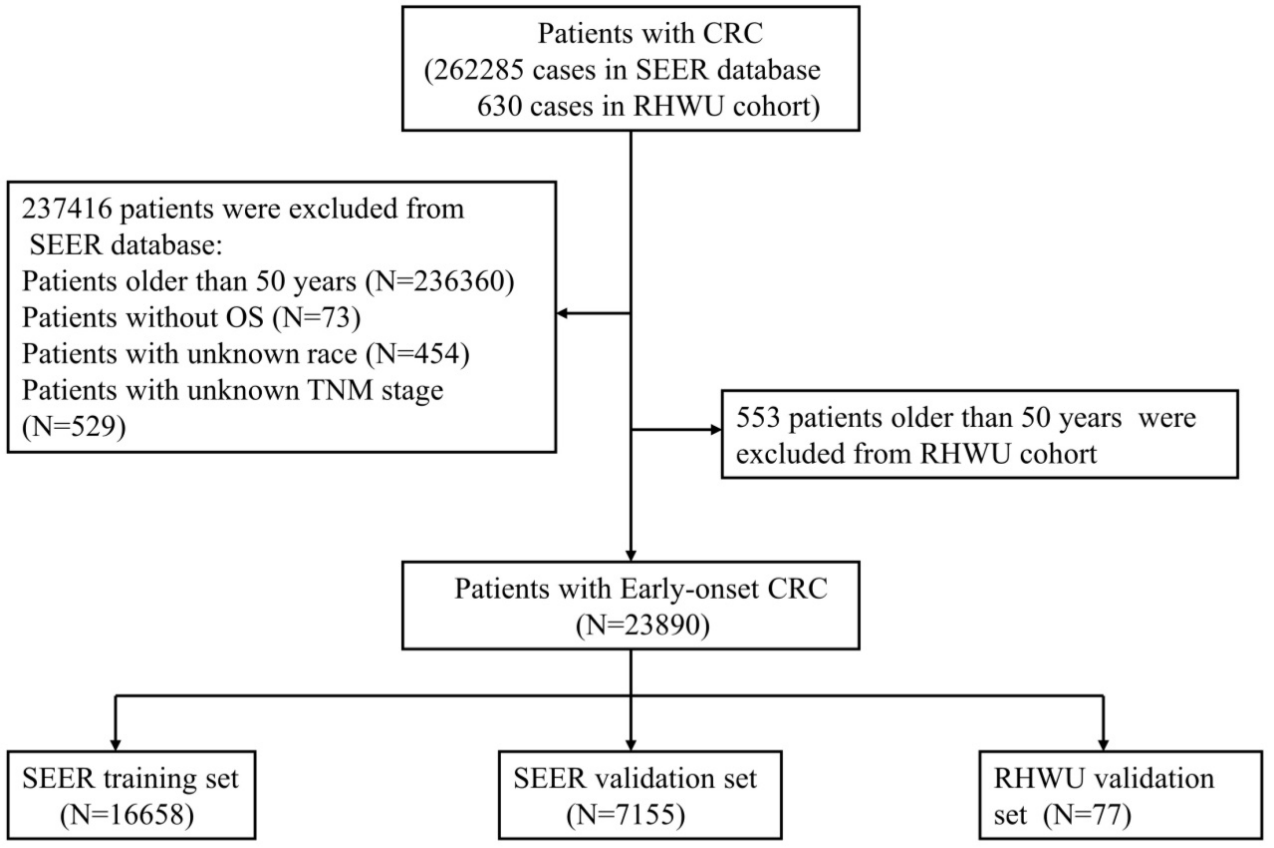
The detailed selection process of patients with EOCRC.

All patients with EOCRC from SEER database between 2010 and 2016 were retrospectively screened. A total of 262285 cases of CRC patients from SEER database were initially reviewed and 23813 patients with EOCRC were finally included into this study. The included patients were randomly assigned to SEER training set and SEER validation set according to the ratio of 7:3. Furthermore, patients' with EOCRC from RHWU was used as an external validation set to detect the generalizability of the survival nomogram. A total of 630 cases of CRC diagnosed between January 2015 and January 2020 were initially screened from RHWU and only 77 patients with EOCRC were finally incorporated into the RHWU validation set. The detailed selection process of EOCRC patients in both databases is shown in Figure 1. This study plan was checked before the initiation of the study by clinical institutional ethics board of our hospital.

#### Inclusion criteria

- Confirmed to be diagnosed with CRC according to histopathology;
- Under the age of 50;
- Patients with overall survival (OS) data.

#### Exclusion criteria

- Younger than 18 years old;
- Complicated with other malignant tumors;
- Loss of vital clinical and survival information.

### Data collection

As our primary goal was to construct a survival nomogram with great practicability, so we retrospectively collected variables that were easily obtained in SEER database and our hospital. The following information of EOCRC patients was abstracted from databases: age, gender, primary tumor site, race, tumor grade, summary stage, TNM stage, T stage, N stage, M stage, tumor size and perineural invasion. Furthermore, we also obtained the therapeutic and survival data of the included patients, such as the surgery for primary site, radiotherapy, chemotherapy, OS.

### Construction and validation of the survival nomogram

SEER training (N=16658) set was employed to create a survival nomogram. LASSO Cox regression analyses were applied to screen clinical features significantly related to OS. Statistically significant features by LASSO regression and clinically informative variables were required for final inclusion into the survival nomogram. Based on the final results of LASSO Cox regression, a survival nomogram including all the independent prognostic parameters was developed for prediction of 1-year, 3-year and 5-year OS. C‐index and calibration curves were exploited to evaluate the discriminative and calibration abilities of the survival nomogram. Time-dependent receiver operating characteristic (tdROC) analyses were performed to assess the predictive accuracy of the survival nomogram, and decision curve analysis (DCA) curve was plotted to further evaluate its clinical utility. SEER and RHWU validation sets were used to determine the internal and external validities of the survival nomogram. In order to compare the predictive performance of the survival nomogram with TNM stage, we divided the patients from SEER database into four groups based on the quartiles of predicted probability 20.

### Statistical analysis

All statistical analyses were implemented with SPSS 22.0 and R 3.4.3 software. The categorical data were represented as number with percentage and tested with Chi-square, while continues variables were expressed as mean with standard deviation and examined by variance analysis. In addition, for the comparison of endoscopic treatment and surgical resection in early-stage (Tis-T1N0M0) patients with EOCRC, propensity score matching 21 (PSM) was performed to balance the basic features between two groups. *P* value less than 0.05 at two sides was viewed as statistically significant.

## Results

### Patients' baseline features

As displayed in Figure 1, a total of 23813 patients with EOCRC meeting the inclusion criteria from SEER database were identified into this analysis and randomly divided into the SEER training set (N=16658) and SEER validation set (N=7155). Moreover, 77 cases of patients with EOCRC from our hospital were included and used as an external validation set. As shown in Table 1, the mean age of EOCRC patients was 42.4 ± 6.2 (years) in the SEER training set, 42.4 ± 6.2 (years) in the SEER validation set, 43.2 ± 4.9 (years) in the RHWU validation set, and no significant differences were observed among the three sets as detected by analysis of variance (*P*=0.396). Moreover, the median survival time was 27.0 (12.0, 50.1) months in the SEER training set, 27.1 (12.0, 50.0) months in the SEER validation set and 45.0 (40.0, 47.0) months in the RHWU validation set. Other clinical and pathological features were listed in Table 1.

### Construction and verification of the survival nomogram in SEER database

**Figure 2:**
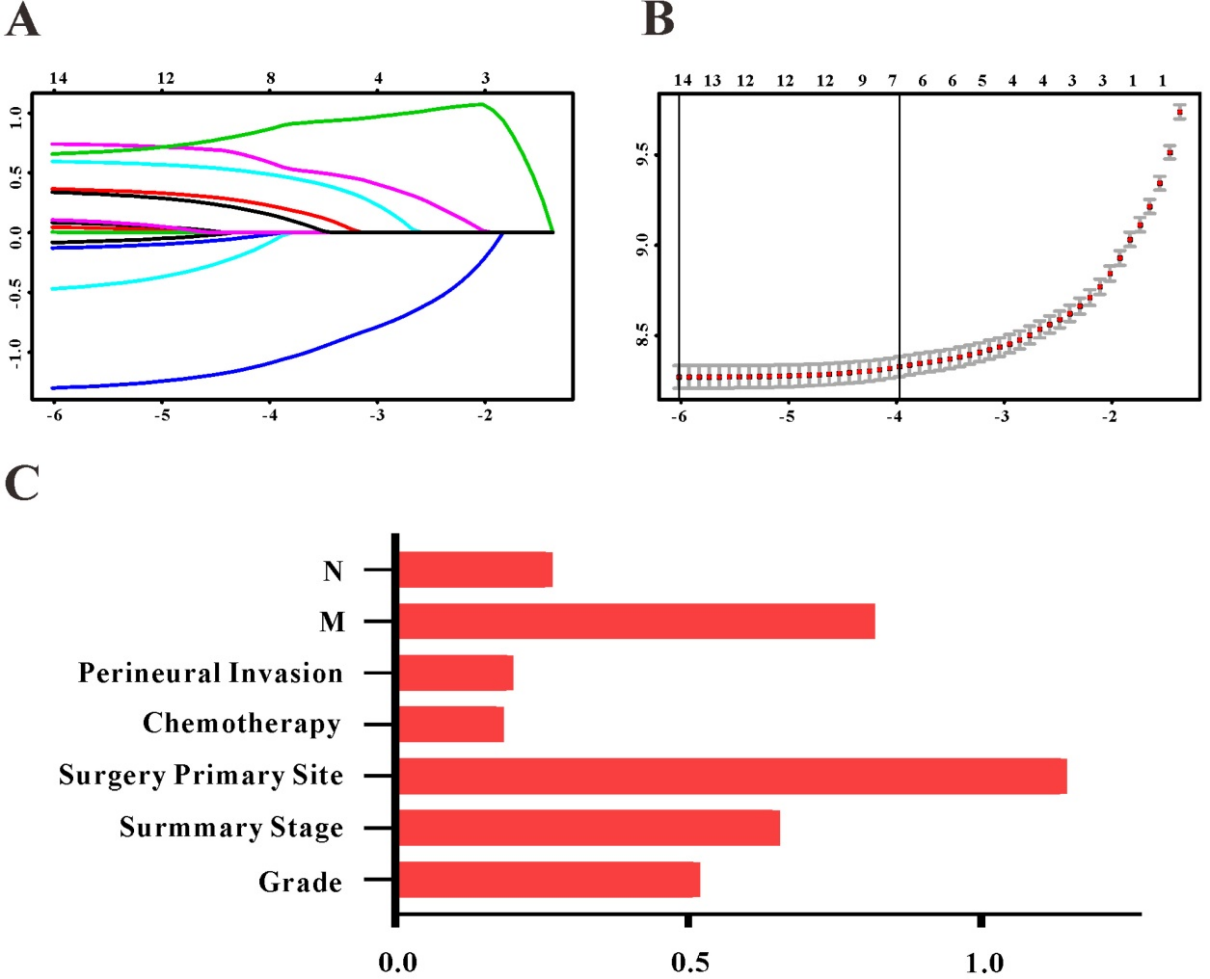
Selection of informative features using LASSO binary Cox regression model. (**A**) Profiles of LASSO coefficient for clinical and pathological features. (**B**) Identification of tuning parameter (λ) in the LASSO Cox model. (**C**) Each horizontal line represents a factor selection result for overall survival. Histogram shows the coefficients of individual features that contribute to the survival nomogram.

**Figure 3:**
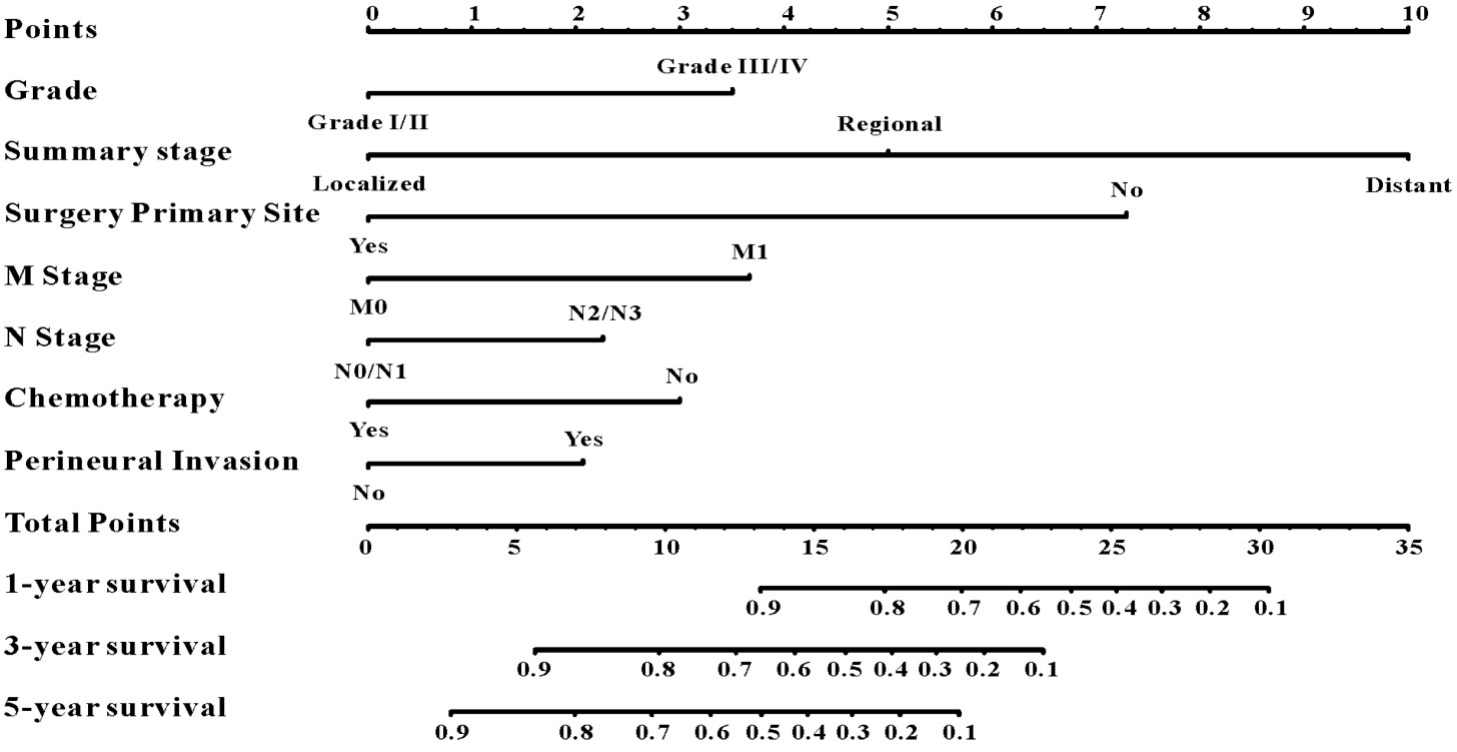
A survival nomogram for predicting 1-year, 3-year and 5-year survival rates of EOCRC patients.

**Figure 4:**
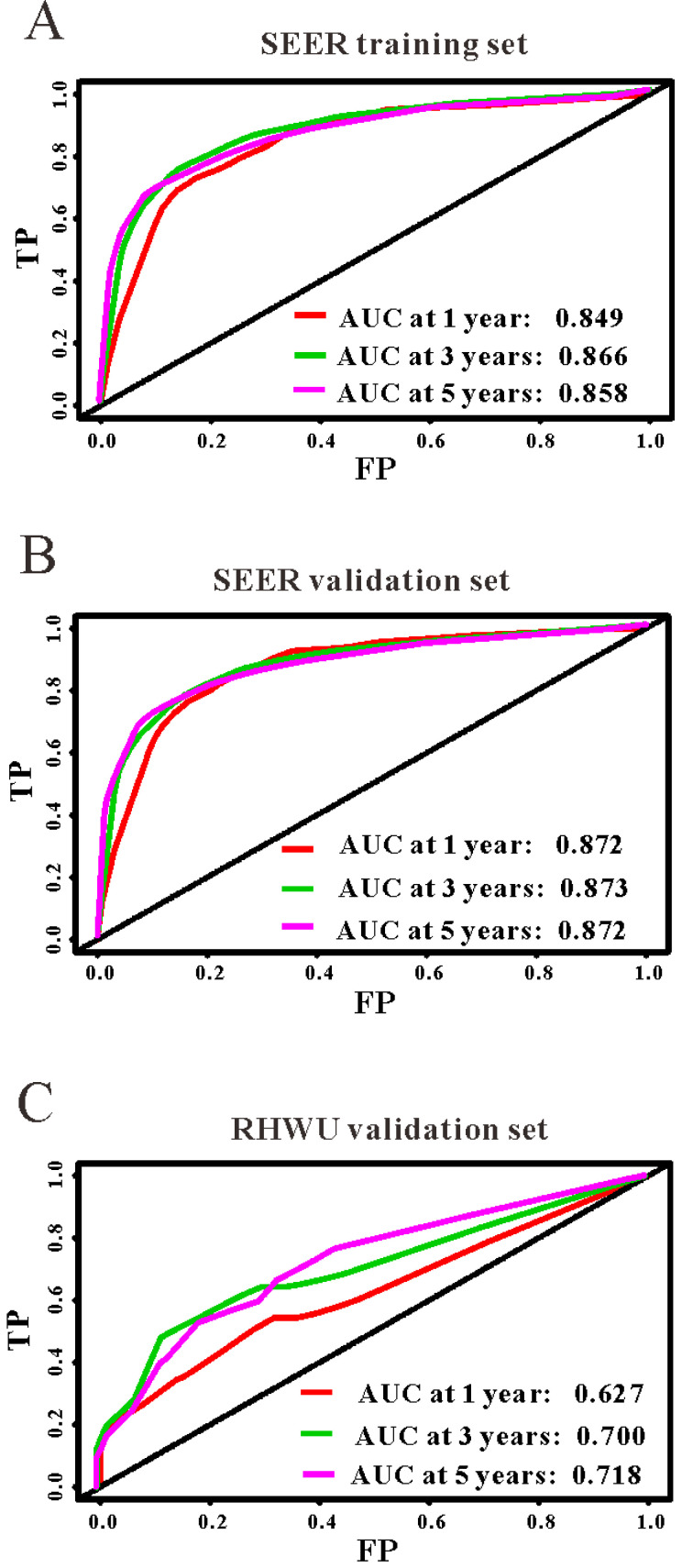
The predictive performances of the survival nomogram for predicting 1-year, 3-year and 5-year overall survival in EOCRC. ROC curves displayed that this survival nomogram discriminated well in SEER training set (**A**), SEER validation set (**B**) and RHWU validation set (**C**).

**Figure 5:**
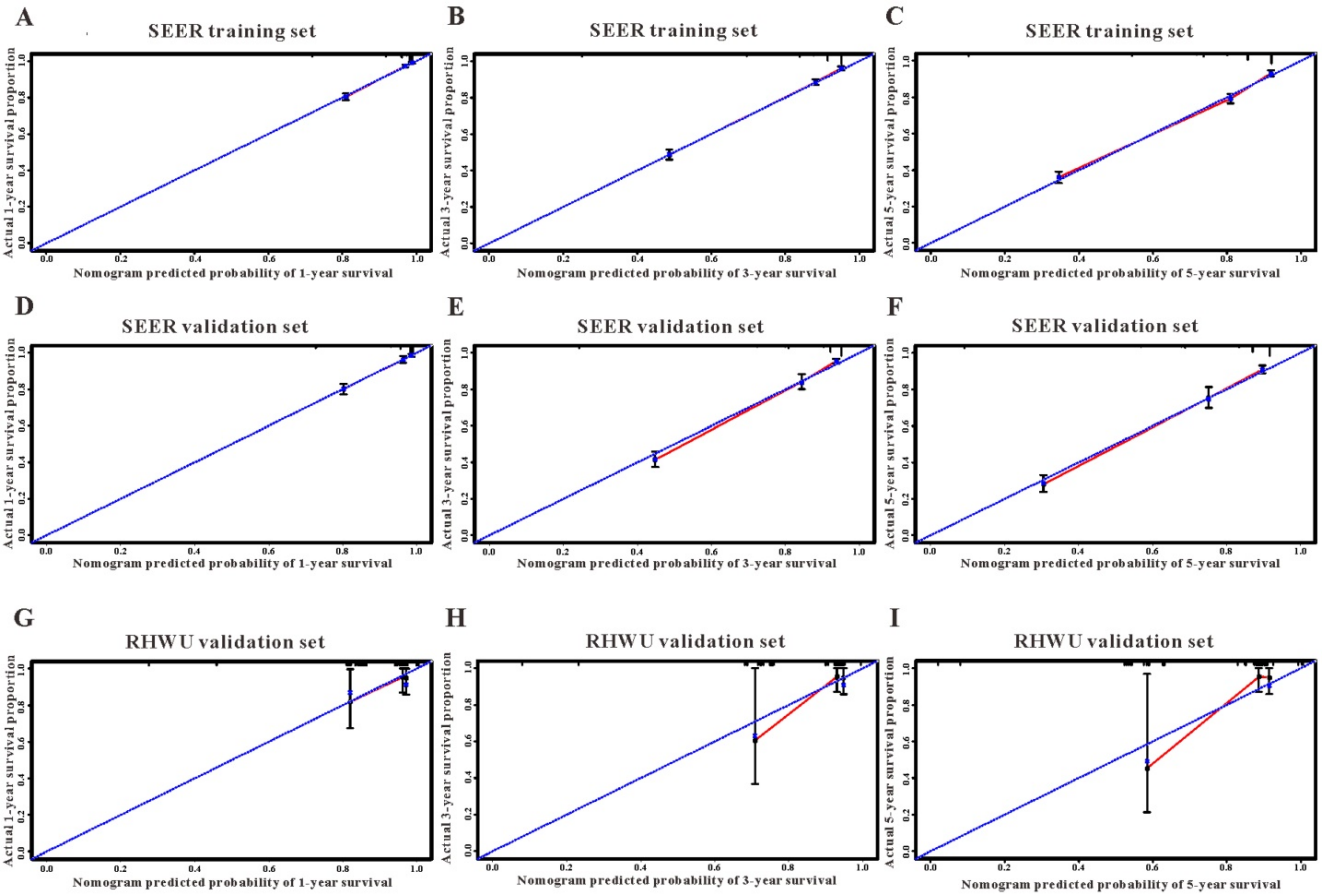
The calibration curves for predicting overall survival in SEER and RHWU sets. (**A-C**) Calibration plots of 1-year, 3-year and 5-year mortality in SEER training cohort; (**D-F**) calibration plots of 1-year, 3-year and 5-year mortality in SEER validation cohort; (**G-I**) calibration plots of 1-year, 3-year and 5-year mortality in RHWU validation cohort.

**Figure 6:**
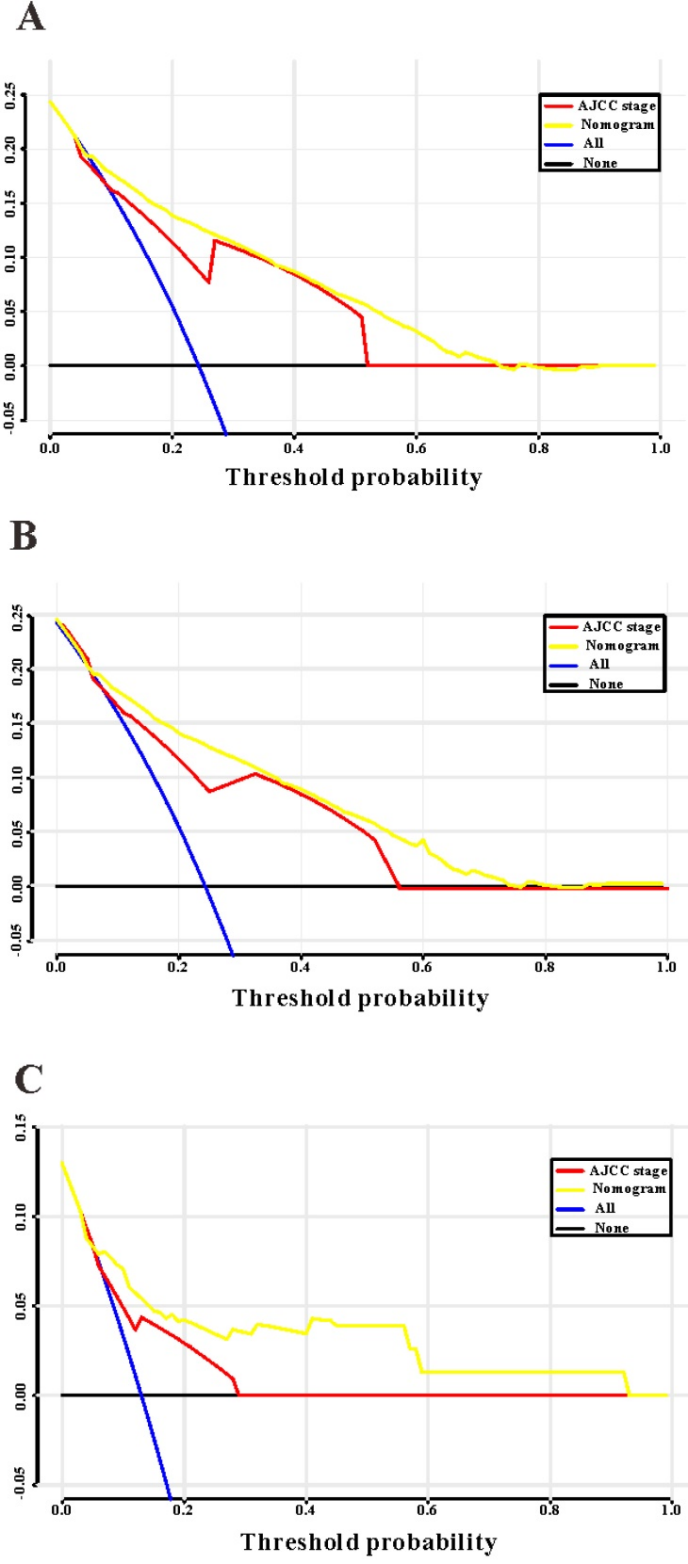
Decision curves analysis (DCA) for the survival nomogram and TNM stage to predict overall survival. (**A**) The DCA of nomogram and TNM stage for overall survival in SEER training cohort; (**B**) the DCA of nomogram and TNM stage for overall survival in SEER validation cohort; (**C**) the DCA of the survival nomogram and TNM stage for overall survival in RHWU validation cohort.

Based on the results of LASSO regression (Figure 2), seven features (N stage, M stage, perineural invasion, chemotherapy, surgery primary site, summary stage and tumor grade) statistically associated with OS were finally incorporated into the development of a survival nomogram in the SEER training set. As displayed in Figure 3, this survival nomogram was very intuitive to predict the 1-year, 3-year and 5-year survival rates of patients with EOCRC. In the SEER training set, the predictive ability of the survival nomogram as measured by C‐index to predict OS was 0.829 (95% CI, 0.821-0.837). More specifically, tdROC analyses (Figure 4A) revealed that the survival nomogram could accurately predicted the 1-year (AUC=0.849), 3-year (AUC=0.866) and 5-year (AUC=0.858) survival rates in patients with EOCRC. Figure 5A-C exhibited the calibration curves of the survival nomogram; plots were very close to the 45‐degree line, indicating that the survival nomogram was well calibrated in the SEER training set. DCA curve also demonstrated that the survival nomogram derived from the SEER training set was clinically useful (Figure 6A).

Similarly, the survival nomogram also obtained good discrimination as demonstrated by C-index of 0.841 (95%CI=0.829-0.853) to predict OS in patients with EOCRC in the SEER validation set. Specifically, tdROC curves (Figure 4B) displayed that the survival nomogram possessed excellent predictive performances for 1-year, 3-year and 5-year survival, as reflected by an AUC of 0.872, 0.873 and 0.872, respectively. To evaluate the calibration of the survival nomogram, we compared the predicted 1-year, 3-year and 5-year survival probabilities with the correspondingly actual observations. As shown in Figure 5D-F, the calibration curves of the survival nomogram exhibited good concordance between the predicted probabilities and actual outcomes. Additionally, our DCA curve from the SEER validation set also proved that the survival nomogram was clinical utility (Figure 6B).

### External validation of the survival nomogram with RHWU cohort

To detect the external validity of the survival nomogram, a cohort of 77 patients with EOCRC from our hospital served as RHWU validation set. The survival nomogram demonstrated an acceptable accuracy with the C-index of 0.796 (95%CI=0.621-0.970) in predicting OS among EOCRC patients. Moreover, tdROC curves (Figure 4C) were plotted to evaluate predictability of the survival nomogram at different time pints and the results were also encouraging. Subsequently, the high-quality calibration curves (Figure 5G-I) showed that the 1-year, 3-year and 5-year survival rates calculated by the survival nomogram were very consistent with the actual observations. The DCA results also demonstrated that the survival nomogram exhibited a favorable clinical applicability (Figure 6C).

### Comparison of the survival nomogram with TNM stage

**Figure 7:**
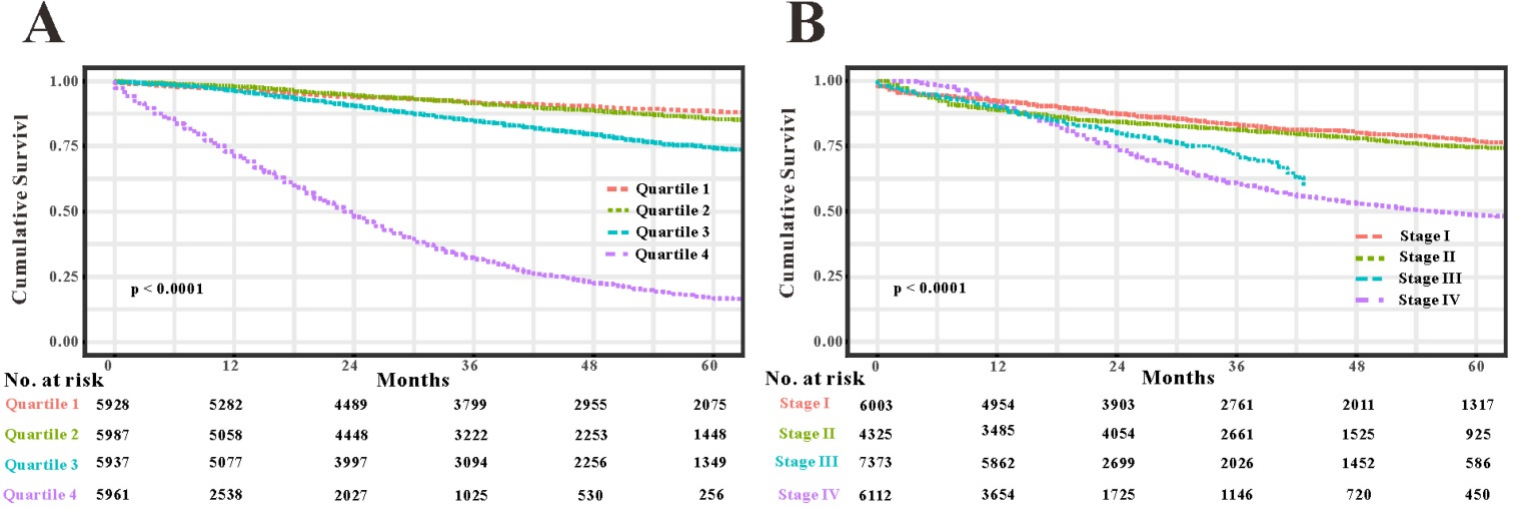
Compare the predictive accuracy of the survival nomogram with TNM stage. (**A**) Kaplan-Meier curves of the quartiles of EOCRC patients stratified by the survival nomogram predicted probabilities in SEER database. (**B**) Kaplan-Meier curves of four groups divided by each TNM stage.

As TNM stage was commonly applied in clinical practice to estimate the prognosis in patients with CRC, so we compared predictability of the survival nomogram with TNM stage. TNM stage obtained acceptable predictive performances as indicated by C-index of 0.774 (95%CI=0.765-0.783) in the SEER training set, 0.777 (95%CI=0.764-0.800) in the SEER validation set and 0.693 (95%CI=0.507-0.808) in the RHWU validation set, respectively. Based on the C-index, we could conclude that the survival nomogram possessed significantly higher C-index than TNM stage. Furthermore, we used the quantiles of predicted probability in SEER database as the cutoff value to divide the patients with EOCRC into four groups. Kaplan‐Meier survival curves were generated to assess differences among the quantiles (Figure 7A) and each TNM stage (Figure 7B). Subsequently, integrated discrimination improvement (IDI) together with net reclassification index (NRI) were introduced into our analysis to compare the prediction efficiency of the survival nomogram and TNM stage in the SEER training set. As illustrated in Table 2, the addition of the survival nomogram could greatly improve the risk reclassification of OS over TNM stage. Taken together, we confirmed that discrimination ability of the survival nomogram was superior to TNM stage in predicting the prognosis of patients with EOCRC.

### Endoscopic treatment versus surgery for early-stage patients with EOCRC

**Figure 8:**
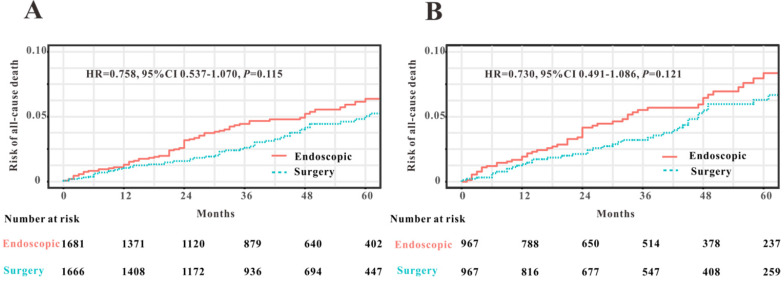
Comparison of overall survival in early-stage patients with EOCRC undergoing endoscopic resection and surgery before PSM (**A**) and after PSM (**B**).

To compare the efficacy of endoscopy and surgical resection for the treatment of early-stage patients with EOCRC from SEER database, we initially applied PSM analysis to balance the clinical features between endoscopy versus surgery groups. A total of 3591 EOCRC patients with early stage (Tis-T1N0M0) were initially screened and 3347 early-stage patients with EOCRC were finally included into this PSM analysis. Four variables were selected for PSM, including age, gender, race and primary site. We found that all the clinical variables were not statistically different between two groups (Table 3). Then Kaplan-Meier plotters were drew to assess the association between two therapy methods and OS (Figure 8). However, the log rank test revealed that no significant association between two therapy methods and OS was observed before PSM (HR=0.758, *P*=0.115) and after PSM (HR=0.730, *P*=0.121). Hence, we could conclude that endoscopic resection possessed comparable OS for the treatment of EOCRC patients with early stage (Tis and T1N0M0) to those treated by surgical resection.

## Discussion

In this study, we identified seven clinical variables significantly related to OS. We constructed and internally validated a survival nomogram based on SEER database, and this nomogram exhibited good discrimination and calibration capabilities in predicting OS of patients with EOCRC. Moreover, RHWU cohort was used as an external validation set and our nomogram also performed well in predicting OS in this population. Finally, this survival nomogram outperformed TNM stage in prediction of OS, implying that our nomogram could facilitate the clinical evaluation of prognosis among EOCRC patients. As far as we concerned, this survival nomogram is the first reported in the literature for accurately predicting OS with good calibration ability in patients with EOCRC.

Although patients with EOCRC possess poor histology and tumor-metastasis predisposition 22, there is no definite conclusion whether patients with EOCRC display worse prognosis compared with traditional CRC patients. Andrea et al. 22 proposed that EOCRC patients exhibited a lower risk of CRC-specific death than patients with late-onset CRC after adjusting for certain clinical features. While, a comparative study 23 based on SEER database from 1991 to 1999 revealed that patients with EOCRC had similar 5-year CRC-specific survival in contrast to patients with late-onset CRC. Abdelsattar et al. 24 undertook a retrospective cohort study based on SEER database from 1999 to 2011 and they found that patients with EOCRC achieved more favorable CRC-specific survival than those with late-onset CRC. However, no study has specifically focused on the independent risk factors of OS in patients with EOCRC. Hence, we undertook this clinical study based on SEER database from 2010-2016, and identified seven informative features significantly associated with OS of patients with EOCRC. More importantly, we created and verified the first survival nomogram devoted to accurately predict OS in patients with EOCRC. In addition, our survival nomogram exhibited good predictive ability for OS in EOCRC patients (C-index of 0.829, 0.841 and 0.796 in the SEER training, SEER validation and RHWU validation sets, respectively), which was much higher than predictive value (C-index less than 0.75) of the nomogram for OS in traditional CRC patients 25-28.

TNM stage is a most commonly used staging system for CRC 29-31, and thus we compared some predictive indexes of the survival nomogram in the SEER database and RHWU cohort with that of TNM stage. We observed that the survival nomogram achieved better predictive efficiency as measured by C-indexes, IDI and NRI than TNM stage. Furthermore, we divided the patients with EOCRC in SEER database into four groups according to the predicted probabilities. Unsurprisingly, Kaplan-Meier curves displayed that the differences among four groups divided by the nomogram were more significant compared with that by TNM stage. It is reasonable that the survival nomogram fared better in predicting OS than TNM stage. Previous studies 32-34 reported that some clinical features, such as demographic features, tumor histopathology, therapeutic regimens also played an important role in the evaluation of prognosis in patients with CRC. However, TNM stage did not take these informative factors into account. To the contrary, our survival nomogram integrated seven prognostic factors and could make more personalized prediction for patients with EOCRC.

Unlike other studies related to EOCRC 16,23,24, perineural invasion was first identified as an essential prognostic factor in patients with EOCRC. Perineural invasion refers to the tumor cells surround and infiltrate the nerve by more than 33% 35. Perineural invasion can occur independently when there is no blood or lymph invasion, and sometimes may be the sole metastasis way for CRC 36. Perineural invasion is a clinical sign of cancer metastasis and thus signifies the unfavorable prognosis of patients with CRC 37,38. However, this clinical factor with great prognostic significance has never been explored in patients with EOCRC. This study revealed that the incidence of perineural invasion was 11.1% in patients with EOCRC from the SEER cohort and 16.9% in EOCRC patients from our cohort. More importantly, the LASSO regression analysis demonstrated the important prognostic role in patients with EOCRC.

In this study, we used RHWU cohort as an external validation set to verify the survival nomogram derived from SEER database. External validation is an indispensable step which integrates the nomogram into the different study population 39. External validation could detect the generalizability of the survival nomogram and ultimately avoid poor goodness-of-fit 40. The survival nomogram obtained an acceptable predictive performance in RHWU validation set, indicating that our nomogram possessed favorable generalizability.

Several limitations still exist in the present study. First, the sample size of RHWU validation set in our analysis was relatively small, which limited the generalizability of our survival nomogram. The small sample size of RHWU validation set led to the acceptable rather than excellent predictive performance. Moreover, both the SEER database and RHWU cohort were retrospective studies, and thus inherent biases were more or less unavoidable. Then, some clinical features, such as serum CEA and microsatellite state, are also important in the estimation of survival of EOCRC. Unfortunately, these variables were not accessible from SEER database, which might discount the accuracy of our survival nomogram. Therefore, prospective researches with large study population from multiple hospitals are further necessary to provide more convincing evidences in the future.

## Conclusions

The survival nomogram based on the most accessible clinical features could precisely predict the survival of individual patients with EOCRC, highly outperforming the predictive accuracy of TNM stage. This survival nomogram will contribute to aid oncologists better risk stratification and prognostication for patients with EOCRC.

## Figures and Tables

**Table 1:** Clinical features of EOCRC patients in SEER and RHWU sets

Characteristics	SEER training set (n=16658)	SEER validation set (n=7155)	RHWU validation set (n=77)	*P* value
Age (years)	42.4±6.2	42.4±6.2	43.2±4.9	0.396
Sex, male	8826 (53.0%)	3725 (52.1%)	34 (44.2%)	0.138
**Race**				0.011
White	12327 (74.0%)	5328 (74.5%)	47 (61.0%)	
Black	2385 (14.3%)	981 (13.7%)	13 (16.9%)	
Others	1946 (11.7%)	846 (11.8%)	17 (22.1%)	
**Primary site**				0.429
Colon	10717 (64.3%)	4600 (64.3%)	55 (71.4%)	
Rectum	5941 (35.7%)	2555 (35.7%)	22 (28.6%)	
**Grade**				0.670
Grade I/II	13978 (83.9%)	5971 (83.5%)	65 (84.4%)	
Grade III/IV	2680 (16.1%)	1184 (16.5%)	12 (15.2%)	
**Summary stage**				0.216
Localized	5680 (34.1%)	2457 (34.3%)	31 (40.3%)	
Regional	6483 (38.9%)	2837 (39.7%)	30 (39.0%)	
Distant	4495 (27.0%)	1861 (26.0%)	16 (20.8%)	
**TNM stage**				0.724
Stage I	4221 (25.3%)	1782 (24.9%)	11 (14.3%)	
Stage II	2992 (18.0%)	1333 (18.6%)	20 (26.0%)	
Stage III	5130 (30.8%)	2243 (31.3%)	30 (39.0%)	
Stage IV	4315 (25.9%)	1797 (25.1%)	16 (20.8%)	
**T stage**				<0.001
T0/Tis/T1/T2	6645 (39.9%)	2820 (39.4%)	12 (15.6%)	
T3/T4	10013 (60.1%)	4335 (60.6%)	65 (84.4%)	
**N stage**				<0.001
N0/N1	13796 (82.8%)	5907 (82.6%)	31 (40.3%)	
N2/N3	2862 (17.2%)	1248 (17.4%)	46 (59.7%)	
**M stage**				0.208
M0	12344 (74.1%)	5358 (74.9%)	55 (80.5%)	
M1	4314 (25.9%)	1797 (25.1%)	22 (19.5%)	
**Tumor size**				<0.001
<1 cm	15810 (94.9%)	6797 (95.0%)	41 (53.2%)	
≥1 cm	848 (5.1%)	358 (5.0%)	36 (46.8%)	
**Surgery for primary site**	13642 (81.9%)	5966 (83.4%)	67 (87.0%)	0.012
**Radiotherapy**	4086 (24.5%)	1775 (24.8%)	1 (1.3%)	<0.001
**Chemotherapy**	10219 (61.3%)	4361 (61.0%)	7 (9.1%)	<0.001
**Perineural invasion**	1845 (11.1%)	796 (11.1%)	13 (16.9%)	0.270
**Survival months median (IQR)**	27.0 (12.0, 50.1)	27.1 (12.0, 50.0)	45.0 (40.0, 47.0)	0.156

EOCRC: early-onset colorectal cancer; TNM: tumor-node-metastasis; IQR: interquartile range.

**Table 2:** Risk reclassification of overall survival by IDI and NRI in SEER training set

Outcome	AUC				IDI		Total continuous NRI	
	Biomaker	Biomaker+clinical model	Clinical model^a^	*P* value^b^	Value (95%CI)	*P* Value	Value (95%CI)	*P* Value
Nomogram	0.829	0.831	0.573	<0.001	0.399 (0.382-0.415)	<0.001	0.573 (0.554-0.595)	<0.001
TNM Stage	0.774	0.786	-	<0.001	0.333 (0.314-0.351)	<0.001	0.488 (0.461-0.510)	<0.001
Nomogram +TNM	0.834	0.837	-	<0.001	0.463 (0.425-0.517)	0.002	0.604 (0.587-0.622)	<0.001

AUC, area under the receiver-operating characteristic curve; IDI, integrated discrimination improvement; NRI, net reclassification index; OS, overall survival; TNM, tumor-node-metastasis;^a^The clinical model for predicting OS are composed of age, gender, race, primary site, T stage, radiation and tumor size; ^b^Biomarker +clinical model versus clinical model.

**Table 3:** Characteristics of early-stage patients with EOCRC before and after matching

Characteristics	Before matching			After matching		
	Surgical resection	Endoscopy	*P* value	Surgical resection	Endoscopy	*P* value
No. of patients	1666	1681	-	967	967	-
Age at diagnosis	42.8±6.0	41.8±6.8	<0.001	42.4±6.6	42.6±6.3	0.371
**Sex**			0.790			0.237
Male	806 (48.4)	821 (48.8)		464 (48.0)	490 (50.7)	
Female	960 (51.6)	860 (51.2)		503 (52.0)	477 (49.3)	
**Race**			<0.001			0.641
White	1281 (76.9)	1153 (68.6)		737 (76.2)	715 (73.9)	
Black	213 (12.8)	300 (17.8)		99 (10.2)	157 (16.2)	
Others	172 (10.3)	228 (13.6)		131 (13.5)	95 (9.8)	
**Origin recode**			0.165			0.136
Hispanic or Latino	255 (15.3)	287 (17.1)		166 (17.2)	142 (14.7)	
Not Hispanic or Latino	1411 (84.7)	1394 (82.9)		801 (82.8)	825 (85.3)	
**Primary site**			<0.001			0.290
Colon	1204 (72.3)	588 (35.0)		544 (56.3)	567 (58.6)	
Rectum	462 (27.7)	1093 (65.0)		423 (43.7)	400 (41.4)	
**Grade**			<0.001			0.058
G1/G2	1550 (93.0)	1628 (96.8)		902 (93.3)	920 (95.1)	
G3/G4	116 (7.0)	53 (3.2)		65 (6.7)	47 (4.9)	
**T stage**			0.001			0.651
Tis	205 (12.3)	339 (20.2)		194 (20.1)	230 (23.8)	
T1A	1298 (77.9)	1149 (68.4)		689 (71.3)	628 (64.9)	
T1B	163 (9.8)	193 (11.5)		84 (8.7)	109 (11.3)	
**Tumor size**			0.001			0.057
<1 cm	1635 (98.1)	1671 (99.4)		948 (98.0)	958 (99.1)	
≥1 cm	31 (1.9)	10 (0.6)		19 (2.0)	9 (0.9)	
**Radiation**			<0.001			0.071
Yes	112 (6.7)	39 (2.3)		55 (5.7)	38 (3.9)	
No/unknown	1554 (93.3)	1642 (97.7)		912 (94.3)	929 (96.1)	
**Chemotherapy**			<0.001			0.079
Yes	143 (8.6)	50 (3.0)		68 (7.0)	49 (5.1)	
No/unknown	1523 (91.4)	1631 (97.0)		899 (93.0)	918 (94.9)	
**Perineural Invasion**			0.093			0.414
Yes	7 (0.4)	2 (0.1)		4 (0.4)	2 (0.2)	
No/unknown	1659 (99.6)	1679 (99.9)		963 (99.6)	965 (99.8)	
**Survival months**	41.0 (20.0, 61.0)	37.0 (17.0, 58.0)	0.008	41.0 (20.0, 61.0)	38.0 (17.0, 59.0)	0.066
**Death**	58 (3.5)	73 (4.3)	0.199	43 (4.4)	56 (5.8)	0.180

## Abbreviations

EOCRC: early-onset colorectal cancer
LASSO: least absolute shrinkage and selection operator
OS: overall survival
SEER: surveillance, epidemiology and end results
TNM: tumor-node-metastasis
IQR: interquartile range
AUC: area under the curve
IDI: integrated discrimination improvement
NRI: net reclassification index
